# Vascular compliance and left ventricular compliance cross talk: Implications for using long-term heat acclimation in cardiac care

**DOI:** 10.3389/fphys.2023.1074391

**Published:** 2023-03-07

**Authors:** Michal Horowitz, Yonathan Hasin

**Affiliations:** ^1^ Laboratory of Environmental Physiology, Faculty of Dentistry, The Hebrew University of Jerusalem, Jerusalem, Israel; ^2^ Adelson Medical school Ariel University, Ariel, Israel

**Keywords:** heat acclimation, intravascular volume, cardiac compliance, HFPEF, cellular signaling

## Abstract

1) The first evidence of the beneficial impact of Long-Term-Heat-Acclimation (LTHA) on cardio-vascular compliance was the positive inotropic response and improved left ventricular (LV) compliance noted when isolated hearts from LTHA rats were studied. Human echo study demonstrates that passive HA affects the right ventricle and the atria as well. 2) There is a cross-talk between vascular and cardiac compliance. Vascular compliance *per se* is defined by central venous pressure—Blood volume relationship—Global Vascular Compliance (GVC). It is determined by the sum of the vascular compliance of the vessels in every organ in any physiological state, varies with LTHA and thus influences cardiac performance. LTHA improves endothelial function, increases NO (nitric oxide) production, in-turn stimulating alterations in ECM (extracellular matrix) *via* the TGF β1-SMAD pathway. 3) LTHA is associated with transformation from fast to slow myosin, heat acclimation ischemic/hypoxic cross-tolerance and alterations in the extracellular matrix. 4) A human translational study demonstrated improved LV compliance following bypass surgery in LTHA subjects compared to controls. 5) Diastolic dysfunction and the impact of comorbidities with vascular and non- vascular origins are major contributors to the syndrome of heart failure with preserved ejection function (HFPEF). Unfortunately, there is a paucity of treatment modalities that improve diastolic dysfunction. 6) In the current mini-review we suggest that LTHA may be beneficial to HFPEF patients by remodeling cardiac compliance and vascular response.

## 1 Introduction

**FIGURE 1:**
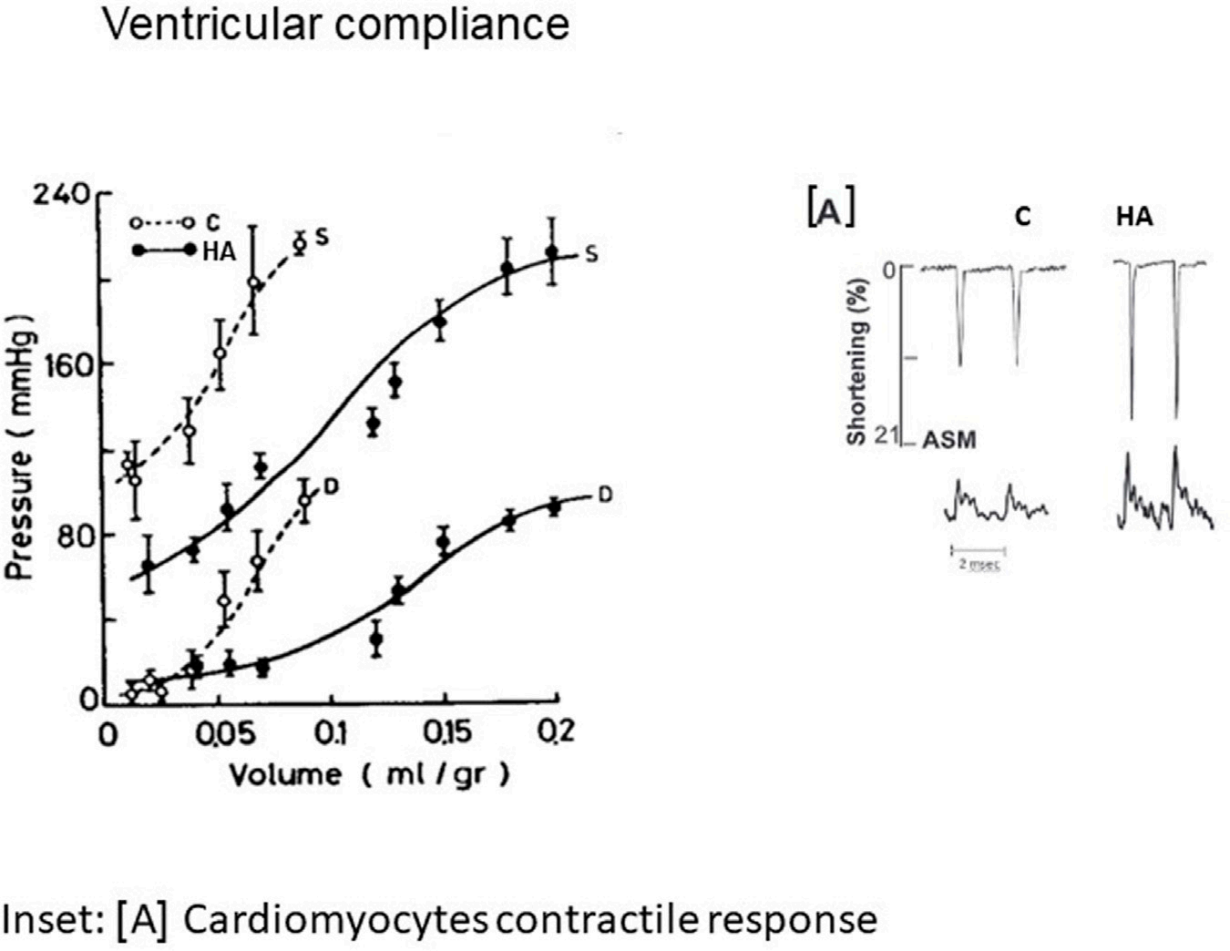
Isovolumic diastolic (D) and systolic (S) volume pressure curve in non-acclimated and 2 m heat acclimated hearts, suggesting enhanced cardiac compliance (with permission of JAP). In the inset [A] individual recordings of cardiomyocytes contractility exhibited by Amplitude systolic motion (ASM) *vs*. Ca^2+^ transient. C-Control; HA-Heat acclimation. With the courtesy of the APS.

Heat acclimation (HA) is achieved *via* exposure to controlled high ambient temperatures under passive (sedentary) or active (with exercise) conditions. Both induce controlled upregulation of body temperature [constant adaptive impulses (Taylor, 2014), initiating pleiotropic adaptive responses facilitating performance in the heat while adjusting body temperature. In the cardiovascular system (CV) this is exemplified by managing the supply of blood to the thermoregulatory organs and the exercising muscles concomitantly. Additionally, the CV system is stressed by demands of coping with alterations in body fluid compartment volumes (e.g., plasma volume expansion vs. preserved extracellular or intracellular compartments) which affect cardiac filling pressure, diastolic volume and compliance (Figure 1).

Elevated stroke volume and enhanced pressure generation, the characteristic signatures of heat-acclimated hearts, are the outcome of physiological changes [e.g., altered Ca^2+^ sensitivity to increase myocyte contraction forces (Cohen et al., 2007; Kodesh et al., 2011) and constitutive cardiac remodeling. Horowitz et al. (1986b) demonstrated that the augmented cardiac chamber compliance of long term heat acclimated (LTHA) rats is due to increased elasticity allowing a significantly greater chamber filling volume without changes in filling pressure. The elegant review by Frangogiannis (2019), emphasizes the importance of the ECM (extracellular matrix) network in maintaining cardiac homeostasis, by providing structural support, facilitating force transmission and transducing key signals to cardiomyocytes, vascular cells, and interstitial cells in health and disease. This cellular signaling (Section 5) is noted in endotherms and ectotherms (Johnston and Gillis, 2022). The timing of these processes upon exposure to chronic heat has not been studied in humans. In ectotherms, the reciprocal ECM changes depend on water temperature. Concurrently, passive myocardial stiffness is strongly related to alterations in the giant myofilament titin, which serves as a spring. In striated muscle the scaffold protein titin is the principal regulator of contractile behavior (Pinzauti et al., 2018; Eldemire et al., 2021). Defining the force and the stiffness of the motor protein myosin, as well as energy utilization or Ca^2+^ sensitivity *via* translation/post translational modifications and phosphorylation of its components (Linke, 2018). Thus, modifications to titin may explain many of the physiological changes observed in the LTHA heart. Evidence of the impact of thermal acclimation on titin in LTHA rats was also noted in trout (Chen et al., 2018). These modifications also explain the findings of O’Neill et al. (2006) regarding the HSP70/slow myosin ratio in the overloaded Plantaris muscle in heat stressed rats, supporting correlations between slow myosin phenotype, thyroxine and HSP70 levels; namely, the factors necessary for enhanced compliance in chronic heat adapted trained rat hearts. Indeed, Horowitz et al. (1986a) demonstrated remodeling to slow myosin phenotype in conjunction with decreased thyroxin levels, inferring that increased energy efficiency is an important adaptive mechanism.

Our human translational study (Pollak et al., 2017) demonstrated that acclimatization to heat protects the left ventricle from increased diastolic chamber stiffness immediately after bypass surgery. The cellular signaling involved was not examined. However, as discussed in Section 2, studies using our experimental animal models allow scrutinization of the mechanisms involved.

The aforementioned beneficial mechanisms observed in the HA heart, both for contractile response and compliance depend on long-exposure. In this mini-review we use an integrative/comparative approach to discuss whether LTHA is beneficial for cardiac patients, particularly those suffering from HFPEF (Heart Failure with Preserved Ejection Fraction). HFPEF is often preceded by, and associated with, diastolic dysfunction.

## 2 Human translational studies on left ventricular compliance and reinforcing animal studies: Impact of heat acclimatization/acclimation and exercise training

In 2017 we published the first human translational study on the impact of heat acclimatization on cardiac compliance post coronary artery bypass surgery (Pollak et al., 2017). We demonstrated that acclimatization to heat protects the left ventricle of the human heart from increased diastolic chamber stiffness immediately after surgery. The human study took place following investigations on the influence of LTHA on the performance of isolated, perfused, rat hearts. The evidence of the impact of LTHA on cardiac compliance accumulated across several studies: 1) Horowitz et al., (1986a), Horowitz et al., (1986b) demonstrated increased compliance, reduced stiffness and transformation from the fast to slow myosin isoform, 2) Levi et al. (1993), Levy et al. (1997) revealed protective features during ischemia and reperfusion, possibly *via* energy-sparing mechanisms, shorter duration of ischemic contracture and energy salvage on reperfusion. These findings were the foundation of the concept of cross-tolerance between LTHA and cardiac ischemic protection. During coronary artery bypass surgery there is severe myocardial ischemia followed by reperfusion.

Both ischemia and reperfusion cause diastolic dysfunction (Levi et al., 1993). Our intraoperative study measuring the relationship between pressure and volume using transesophageal echocardiography during volume load showed that LTHA provides protection from an increase in diastolic chamber stiffness usually noted immediately after coronary artery bypass surgery and prevents diastolic dysfunction (Pollak et al., 1998). This demonstration of LTHA-ischemic cross tolerance supported our conclusions from animal studies that LTHA increases ventricular compliance.

Recent human translational studies, using 1) Short-term isothermic heat acclimation achieved by cycling at a 32°C and 70% R.H. (Relative Humidity) at an exercise intensity of 2.0–2.7 W kg-1 for 5 days compared to daily cycling for 90 min, in temperate environmental conditions of 21.5°C and 36% R.H. and 2) Passive heat acclimation (12 d, −48°C, 50 min) (Parsons et al., 2020; Wilson et al., 2020) indicated that short-term, active heat acclimation enhances ventricular compliance, along with physiological thermoregulatory responses (e.g., plasma volume expansion, decreased heart rate, altered cardiac filling pressure and increased stroke volume), rather than changes in cellular elasticity. No intrinsic cellular or molecular explanation for these responses were provided. Complementary to these investigations, the comprehensive study by Kodesh et al. (2011) on the structural (echo), electro-physiological (EC-coupling) and transcriptional (molecular pathways) changes observed in rat hearts demonstrated that LTHA with exercise training (compared to passive HA or exercise training alone) is a stress in and of itself, noted by the fact that heat exposure overrides the benefits of exercise training. The LTHA sedentary rats (and the matched exercising rats) had lower contractile kinetics and enhanced ventricular compliance. Furthermore, swimming training (Levi E, MSc Thesis, HU (provided cardio-protection from ischemic damage in the LTHA and non-acclimated trained rats. The acclimated-sedentary and acclimated-trained rats, had significantly lower negative lusitropic (-dP/dt/P) response vs. the pre-treated controls (with similar filling pressures), suggesting decreased ventricular stiffness. The impact of passive LTHA in rats confirms the findings of Horowitz et al. (1986a) but differs from the effects of 5 days of passive HA in humans (Trachsel et al., 2020), where minimal adaptive responses were detected in the heart. While Parsons et al. (2020) and Trachsel et al. (2020), present pre *versus* post treatment comparisons within the same group, we performed within and between (namely, comparisons between all stressors employed) group analyses of the long-term impacts of the stressors, when hormonal responses (e.g., reduced thyroxin) leading to myosin phenotype changes take place (Horowitz, 2003). Notably, the impact of seasonal temperatures on slow myosin were reported in *Camelus dromedarius* (Abdelhadi et al., 2012). In addition to the shift from the fast to slow myosin phenotype, LTHA increases endurance during Ca^2+^ overload (Mirit et al., 2000) and changes the Ca^2+^ transient/amplitude systolic motion ratio (Kodesh et al., 2011). Furthermore (Horowitz et al., 2004; Mreisat et al., 2020), LTHA is also limits the negative impacts of reactive oxygen species (ROS). These physiological markers support our hypothesis that titin and its interconnection with the ECM contribute to the adaptive signaling in the passive and trained LTHA hearts (Kodesh et al., 2011). This will be discussed further in Sections 4 and Sections 5.

## 3 Global vascular compliance (GVC)—Impact of LTHA

**FIGURE 2:**
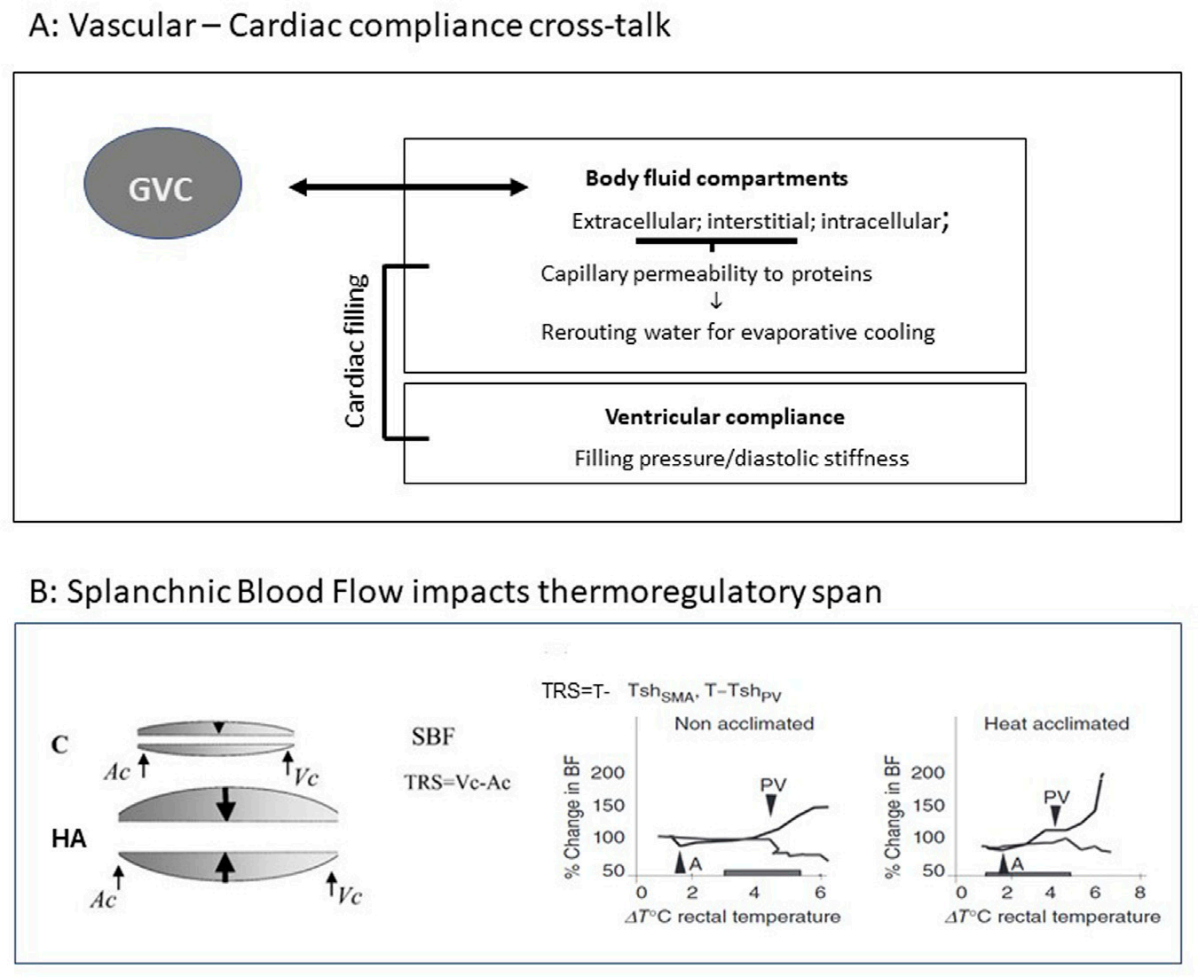
**(A)** Vascular *vs*. ventricular compliance cross talk (GVC). **(B)** Splanchnic Blood Flow impacts thermoregulatory span (TRS). Left: Schematic illustration of splanchnic blood flow (SBF) before and post LTHA. Arrows denote the range of flexibility allowing maintenance of adequate SBF and heat dissipation to the periphery by activation of the major thermoregulatory reflex (splanchnic vasoconstriction *vs*. peripheral vasodilation). Right: Illustration of dynamic TRS. (SMA- super mesenteric artery, PV-portal vein, *Ac* and *Vc*—Arterial and venal constriction (. Rats were subjected to heat stress at 42°C. Pre-acclimation: activation of thermoregulatory reflex: SMA 37.3°C PV40.1°TRS = 2.8°C. Following LTHA; SMA 37.4°C PV41.0°C TRS = 3.6°C. Modifies from Horowitz J. Therm Biology Horowitz (2001) and Horowitz (2014), Comprehensive Physiology 4, 199-230.

Global vascular compliance (defined in this review: GVC, Figure 2) is determined by the △ of central venous pressure—△ blood volume following saline infusion and the ability of the vascular compartment to retain (or drain) the infused volume (Morimoto, 1990). Historically, maintenance of central venous pressure was considered the most important factor controlling cardiac output (Guyton AC, 1963). Nowadays the complexity of this physiological situation is better understood, with advancements in our knowledge since then [as reflected, e.g., (Yoshiga et al., 2019), who compared the impact of Atrial Natriuretic peptide vs. central venous pressure regarding the control of cardiac output during exercise].

Certain situations such as thermal stress or dehydration, cause blood flow redistribution based on vessel compliance, e.g., skin vs. splanchnic vascular beds and thus affect GVC (Morimoto, 1990). Based on Starling equilibrium rules, the altered volumes of body water compartments, namely, intravascular, interstitial, and intracellular, play a major role in the aforementioned relationships. Horowitz (2001) showed that following LTHA water for evaporative cooling was rerouted from the plasma (extracellular compartment) to intracellular compartment. Concomitantly, capillary bed permeability to plasma colloid-osmotic proteins decreased (Horowitz, 2001), suggesting that by manipulating plasma colloid osmotic pressure, vascular and extravascular compartments act as a unified dynamic compartment affecting both vascular/extravascular compliance (Figure 2A). Horowitz et al. (1988) demonstrated that the compliance of the GVC of non-acclimated rats during hyperthermia remained unchanged, whereas a profound decrease was noted in LTHA rats. This finding is not in line with Horowitz and Samueloff (1988) who demonstrated augmented splanchnic blood flow in LTHA. *In-vivo* measurements of superior mesenteric artery (SMA) and portal vein (PV) blood flow (using a transonic blood flow meter) in LTHA heat stressed rats confirmed these findings. PV vasodilation, a marker of the failure of the thermoregulatory reflex, occurs later and at a higher temperature in LTHA than in non-acclimated rats, (Figure 2B), Haddad and Horowitz (1999) [and Horowitz M Figure 1C in Horowitz (2014) showed that LTHA changes the dynamics of the major thermoregulatory reflex and, in contrast to non-acclimated rats, maintains splanchnic perfusion and heat convection to the periphery from the highly metabolic splanchnic area for an extended time (Figure 2B).

The delayed responsiveness of the splanchnic circulation to thermal stress was unknown and not considered in the experimental setup of Horowitz et al. (1988) which preceded Haddad and Horowitz, (1999). The changes in the dynamics of the major thermoregulatory reflex as exhibited in the SMA of the hyperthermic LTHA rats suggests neural control. Likewise, decreased capillary bed permeability to proteins (Horowitz and Samueloff, 1988) indicates that the interstitial space is a major controller of vascular/interstitial GVC. Whether this is due to neural regulation alone or combined with cellular (e.g., endothelial or myocyte) signaling, was unknown at the time, although the use of LNNA, a NO inhibitor supported the involvement of altered endothelial signaling. In a human translational study, Brunt et al. (2016) investigated the impact of 8 weeks of thermal therapy (40.5°C water bath, to maintain a T core of 38.5° for 60 min) and demonstrated improved endothelium-dependent dilatation and decreased arterial stiffness. The role of NO and cellular signaling involved with GVC will be discussed below (Sections 5).

Taken together, our experimental series confirmed that LTHA affects vasculature GVC. Unfortunately, the impact of short-term acclimation on vascular compliance was not examined. Recent studies on the bi-phasic nature of signaling during heat acclimation, show that the initial pathways involved in the maintenance of DNA integrity are replaced by pathways contributing to acclimatory homeostasis (Horowitz, 2017), reinforcing the gradual development of altered GVC related to the cumulative responses of the thermally-activated peripheral vasculature.

## 4 Long- but not short-term acclimation increases cardioprotection

The long period required to achieve acclimatory homeostasis in the heart was shown in several studies. Assayag et al. (2010) demonstrated that HA induced cardio-protection (delayed apoptosis) only occurs following LTHA (33°C–34°C, 30d). Alexander-Shani et al. (2017) revealed a similar pattern for HIF 1α activation and heat acclimation cross-tolerance with ischemia/hypoxia; Cohen et al. (2007) demonstrated the beneficial effect of LTHA vs. short-term on the mechanical properties of cardiomyocytes (e.g., desensitized myofilaments, as indicated by a rightward shift in the ASM-Ca^2+^ relationships, despite no change in SR Ca^2+^ pool size). Notably et al. (2014) reported similar findings regarding neuroprotection mediated by heat acclimation-hypoxia cross-tolerance *via* reduced calcium permeability.

Recently acclimatory homeostasis has been attributed to epigenetic mechanisms such as post translational histone modifications (Tetievsky and Horowitz, 2010) and (Horowitz, 2010; Horowitz, 2017). The correlation between physiology and cell signaling/molecular biology in heat-acclimation highlights the pathways common to this phenomenon in the global vascular response and the heart, e.g., (Haddad and Horowitz, 1999; Assayag et al., 2010; Yacobi et al., 2014; Brunt et al., 2016; Alexander-Shani et al., 2017; Johnston and Gillis, 2022) and others as detailed in Sections 5.

## 5 Cellular signaling associated with vascular and ventricular compliance in long-term- heat-acclimation

The molecular pathways responsible for improved vascular and left ventricular compliance in response to HA have not been thoroughly investigated. However, the known pathways are shared by both organs. Of prime interest are the elegant publications by Johnston and Gillis (2022) who studied rainbow trout and zebrafish [e.g., (Johnson et al., 2014)] and noted that the increase in deposited collagen, a major determinant of elasticity/stiffness of the extracellular matrix in these fish, is reversible and the collagen content changes in response to thermal (cold *vs*. warm) acclimation in order to maintain ventricular performance as the water temperature varies with the seasons. Other studies (Jørgensen et al., 2014; Keen et al., 2016) show comparable adaptive responses, e.g., in ectothermic vertebrates the collagen content decreases as environmental temperature increases. Importantly, mammals have similar cell signaling (e.g., induced by changes in biomechanical forces (Frangogiannis NG. (2019), however these were not studied with respect to chronic heat. Indeed, changing collagen content of ectotherms in response to seasonal temperature variations or thermal acclimation may also stem from altered biomechanical loads.

The ECM is involved in many aspects of cardiovascular physiology and pathology (e.g., (Bloksgaard et al., 2018). Thus, based on the knowledge of cell signaling in mammals (humans, rodents) Johnston and Gillis (2022) examined changes in the ECM in thermally acclimated trout and zebrafish and demonstrated that the TGF β1-SMAD pathway plays a major role. In a human translational study on thermal therapy Lucas et al. (2018) demonstrated that endothelial cells share similar pathways, involving the NO induced TGF β1-SMAD pathway. Similarly, a hamster’s model was used to explain the mechanisms involved in Japanese Waon (soothing warm) therapy for cardiac patients and identified endothelial NO as a fundamental component (Miyata and Tei C, 2010). Haddad and Horowitz (1999) used LNNA (an inhibitor of NO) and confirmed the role of NO and endothelial cells in splanchnic blood flow regulation in the LTHA rats. We have firm physiological evidence that the ECM and titin both play important roles in the impact of LTHA on ventricular compliance. Recently, Paulus and Zile (2021) provided molecular evidence that hemodynamic load–induced alterations in ECM laminin cause changes in titin that affect cardiomyocyte and myocardial stiffness.

## 6 Clinical implications

Heart failure related to myocardial dysfunction causes significant morbidity and mortality. About half of these patients have diastolic dysfunction with preserved systolic function (HFPEF—Heart Failure with Preserved Ejection Fraction) (Adamczak et al., 2020). “HFPEF is often preceded by, and associated with, diastolic dysfunction, which is a consequence of left ventricular wall stiffening and impaired diastolic ventricular filling”. Reduced endothelial function with decreased cardiovascular concordance is very common Brunt and Rossman (2017). A special issue in AJP-heart and circulation (Bloksgaard et al., 2018) was dedicated to the role of the ECM (e.g., the collagen scaffold including collagen types and levels) and the cross talk between cells that modify ECM stiffness. Unfortunately, there are currently very few treatment modalities that stop the deterioration in patients with HFPEF. Given that biomechanical signals are linked to fibrosis, the preliminary study by Edelmann et al. (2011) showed that long-term exercise improves the diastolic function of these patients. In a more recent study Hotta et al. (2017) confirmed this phenomenon in young and aged rats. Furthermore, by using radioactive microspheres to measure blood flow in the coronary circulation they found a correlation between the coronary arteries and aortic distensibility (namely, enhanced compliance increased coronary blood flow). This finding supports our hypothesis regarding cross-talk between vascular and cardiac distensibility with extracellular matrix stiffness as the common denominator. The review by Budde et al. (2022), on HFPEF supports our theories and emphasizes that ROS and Ca^2+^ sensitivity are comorbid factors in HFPEF. The hazards of both ROS and alterations in Ca^2+^ sensitivity decrease upon LTHA (Mreisat et al., 2020). Based on the beneficial effects of LTHA on cardiac and vascular compliance described in the present review, it seems that patients with HFPEF could benefit from heat acclimation, such as from its epigenetic impacts, (e.g., Titin N2B/N2BA (stiff/complaint isoforms) ratio (Li et al., 2022). A recent meta analyses (Ye et al., 2020) also support that long-lasting thermotherapy (Sauna), similarly to LTHA, improves cardiac and vascular function of cardiac patients. The importance of clinical epigenetics as a new tool to be employed for a management of HFPEF is still sparse (Hamdani et al., 2021). The evidence that LTHA an epigenetic phenomenon (Tetievsky and Horowitz, 2010) when linked to human translational clinical study on cardiac diastolic compliance post-surgery (Pollak et al., 2017) indicates its beneficial impact on cardiac patients.

## 7 Summary and perspectives

The findings presented in this mini-review focus on the beneficial effects of LTHA on ventricular and vascular compliance fostering cardioprotection. Our data (from animal models) suggests that epigenetic machinery is involved in the dynamic changes to gene expression in pathways linked to increased muscle efficiency and energy saving, e.g., changes in myosin phenotype, HIF-1α activation and mitochondrial activity (reviewed above). Collectively (Sections 2 and Sections 3) LTHA leads TGF 1β-SMAD activation, perhaps *via* NO and ECM alterations in myocyte born fibroblasts, namely, the fibroblasts that proliferate post damage and closely resemble neonatal cardiac fibroblasts, which form the fibrotic scar in the failing heart (Kretzschmar et al., 2018; Hanna et al., 2021). LTHA achieved *via* continuous prolonged exposure to elevated ambient temperatures is practical for animal model studies but not for humans. Thus, thermal therapy protocols such as those currently used for other vascular problems together with global transcriptome analyses should be considered.

## Abbreviations

Acclimatization, Acclimatization is defined as adaptation to the natural environment whereas Acclimation occurs under artificial pre-set conditions (IUPS Thermal Commission (2001) -. Jpn. J. Physiol. 51, 245–280; GVC, Global Vascular Compliance; Left ventricular compliance, Ventricle pressure vs. filling volume; ECM, Extracellular matrix; Titin, Principal regulator of the contractile behavior of muscle, It defines the force and the stiffness of the motor protein myosin; NO → TGF β1 →-SMAD, This pathway is shared by both endothelial and the cardiac cells, collectively stimulate neovascularization, and suppression of tumor growth factor beta1/Smads signaling; Cardiomyocyte induced fibrblasts, post damage fibroblasts closely resemble neonatal cardiac fibroblasts which form the fibrotic scar; Cross-tolerance, Developing of a tolerance for one stress leads to tolerance of additional stress sharing similar protective pathways; HFPEF, Heart failure with preserved ejection fraction.
